# The Development of an Efficient In Vitro Indirect Regeneration System for *Tibouchina granulosa* (Desr.) Cogn.

**DOI:** 10.3390/plants14172677

**Published:** 2025-08-27

**Authors:** Fei Xiao, Jiemei Yu, Lan Wang, Xinru Qin, Mengjia Wu, Seping Dai, Xiaomei Deng

**Affiliations:** 1 Guangdong Key Laboratory for Innovative Development and Utilization of Forest Plant Germplasm, Guangzhou 510642, China; xiaofei20232023@163.com (F.X.); wlanys0828@163.com (L.W.); 18702612589@163.com (X.Q.); 20242159034@stu.scau.edu.cn (M.W.); 2 College of Forestry and Landscape Architecture, South China Agricultural University, Guangzhou 510642, China; 3 Guangzhou Institute of Forestry and Landscape Architecture, Guangzhou 510095, China; yujiemei2025@163.com

**Keywords:** callus induction, adventitious bud induction, rooting

## Abstract

This study established an efficient in vitro regeneration system for *Tibouchina granulosato* (Desr.) Cogn. petiolated leaves to address the low propagation efficiency and propagatable germplasm shortages. The results revealed that the Murashige and Skoog (MS) medium supplemented with 1.1 mg/L of Zeatin (ZT) and 0.2 mg/L of 1-naphthyl acetic acid (NAA) was the optimal formulation for callus induction, yielding callus induction of 89.59%. For adventitious bud induction, the combination of 2.0 mg/L of 6-benzyladenine (BA) and 0.4 mg/L of NAA proved most effective, achieving an induction rate of 83.33%. Additionally, the adventitious shoots exhibited remarkable elongation when cultured in a medium containing 0.2 mg/L of BA and 0.04 mg/L of NAA. All explants rooted when treated with 0.5 mg/l NAA, inducing a mean number of 6.90 roots per plant and a survival percentage of 91.00%. This study provided technical support for the promotion of superior varieties and genetic improvement of *Tibouchina granulosa*.

## 1. Introduction

*Tibouchina granulosa* (Desr.) Cogn. belongs to the genus *Tibouchina* of the family Melastomataceae, characterized as a perennial plant adapted to hot, humid climates. It thrives in direct sunlight but can tolerate partial shade, exhibiting limited cold tolerance and moderate drought resistance. Well-drained acidic soils are preferred for optimal cultivation [1]. Native to the Atlantic Forest of Brazil, it was introduced to the Guangzhou Forestry and Landscape Science Research Institute in 2006. Typically, the species exhibits bluish-violet flowers, whereas we selected an individual that displays pink blooms within this population, representing a distinct phenotypic difference.

*T. granulosa* is a prized ornamental species characterized by its lustrous emerald-green foliage, adaptable growth habit, and spectacular floral displays. In Guangzhou’s subtropical climate, it blooms biannually, producing dense floral clusters that create breathtaking landscape vistas [2]. Its adaptable growth habit supports diverse horticultural uses, including solitary specimen plantings, clustered garden groupings, and roadside landscape enhancement, highlighting substantial potential for landscape architecture and urban greening projects [3]. Ecologically, the trichomes on adaxial and abaxial leaf surfaces effectively capture airborne particulate matter, reducing localized air pollution [4,5]. This PM sequestration capacity contributes substantially to urban ecosystem services. Moreover, leaf extracts demonstrate potent anti-inflammatory activity [6], while floral tissues contain bioactive alkaloids, flavonoids, phenolic acids, and antioxidant compounds [7]. This multifunctional profile highlights the value of *T. granulosa* in sustainable urban planning and green biotechnology applications.

The population of *T. granulosa* imported to China is currently limited, with only one individual exhibiting pink flowers, rendering the resources extremely scarce. Furthermore, *T. granulosa* exhibits low seed production, and according to Zaia & Takaki’s study, 70–80% of the seeds fail to complete embryo development, resulting in an exceptionally low germination rate [8]. A study found that *T. granulosa* exhibited optimal cutting propagation results when the upper branches were treated with 100 mg/L of NAA for 2 h between May and November. However, treatments conducted at other times proved less effective [9]. These factors significantly limit the rapid propagation of *T. granulosa*.

Plant tissue culture can reduce the impact of environmental factors on plant growth, requires less space, and allows for the rapid production of a large quantity of genetically stable seedlings within a short time [10]. However, to date, no regeneration studies have been reported for *T. granulosa*.

Therefore, this study employs in vitro regeneration technology with petiolated leaves to propagate superior, pink-flowered specimens of *T. granulosa*, aiming to develop low-cost and high-efficiency propagation techniques and simultaneously lay the foundation for future genetic transformation studies of this species.

## 2. Results

### 2.1. Effect of Explant Types on Callus Induction

The callus induction rate and morphological structure varied significantly depending on explant types in *T. granulosa*. As illustrated in Table 1, petiolated leaf explants demonstrated the highest induction rate (75.00%), producing abundant green and compact calluses favorable for subsequent differentiation. Epicotyl explants exhibited the second-highest induction rate (70.83%), generating limited yellow–green callus clusters. Petiole-free leaf segments exhibited the lowest induction efficiency (56.94%), characterized by sparse green callus formation. Comprehensive analysis identified petiolated leaves as the optimal explant for efficient callus induction in *T. granulosa*.

### 2.2. Effect of ZT and NAA Concentration Combinations on Callus Induction

**Figure 1:**
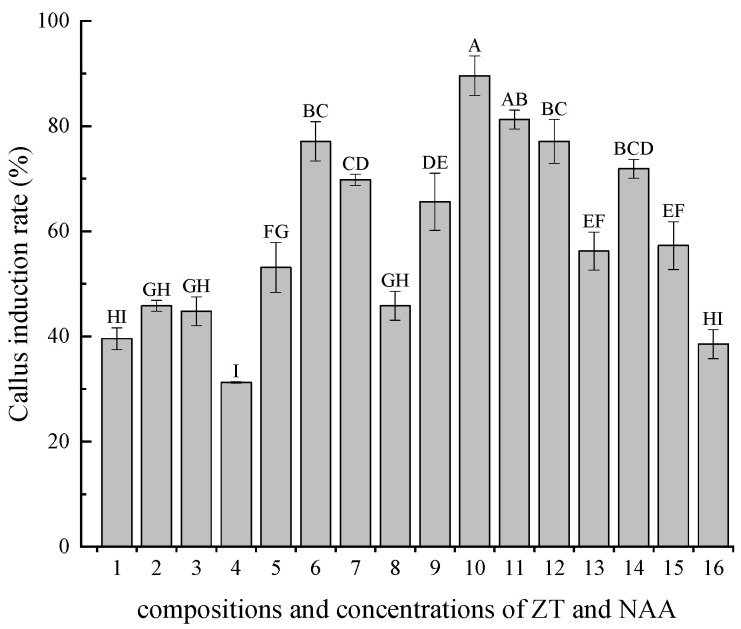
Effect of ZT and NAA concentration combinations on callus induction. Capital letters indicate significant differences (*p* < 0.05) in Duncan’s multiple range test.

**Figure 2:**
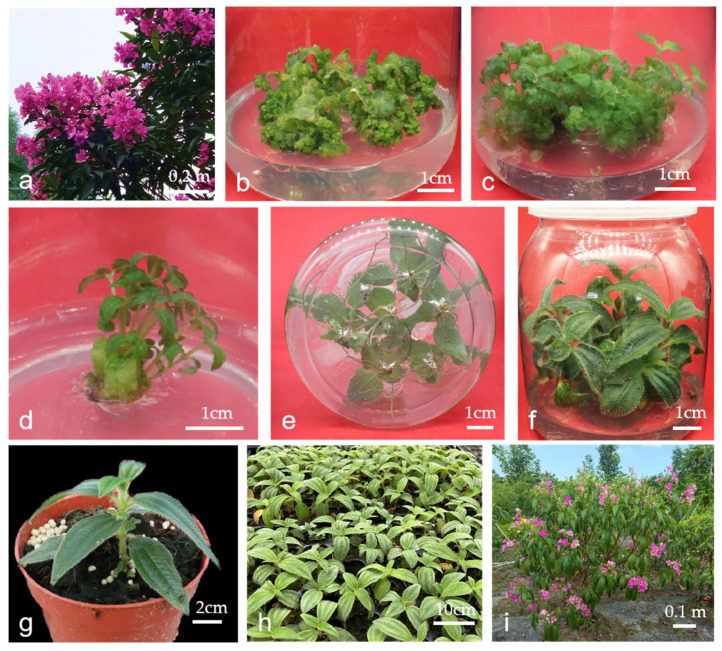
(**a**) *Tibouchina granulosa* (Desr.) Cogn.with pink flowers; (**b**) callus induced by petiolated leaves; (**c**) adventitious bud induction; (**d**) adventitious bud elongation; (**e**,**f**) rooting; (**g**–**i**) transplanting.

Mechanically wounded petiolated leaf explants were aseptically cultured on an MS medium supplemented with varying concentrations of ZT and NAA. Statistical analysis revealed significant effects of growth regulators on callus induction efficiency (Table 2). Maintaining either ZT or NAA at a fixed concentration while altering the concentration of the other hormone resulted in a quadratic trend in the callus induction rate (Figure 1). The comparative evaluation demonstrated that 1.1 mg/L of ZT, combined with 0.2 mg/L of NAA, achieved optimal induction efficiency (89.59%), producing green, compact, nodular callus masses with robust morphogenetic potential (Figure 2b). Conversely, 1.3 mg/L of supraoptimal ZT induced callus browning, particularly at NAA concentrations of 0.3–0.4 mg/L. Suboptimal ZT at a concentration of 0.8 mg/L generated non-proliferative white tissues. At reduced cytokinin/auxin ratios (a ZT concentration of 0.8 mg/L with an NAA concentration of 0.3–0.4 mg/L), the callus differentiated into a limited number of adventitious roots. Conversely, with elevated cytokinin/auxin ratios (ZT at a concentration of 1.0~1.1 mg/L with NAA at a concentration of 0.1~0.3 mg/L), the callus transformed into compact, green, nodular structures capable of inducing adventitious buds. Thus, the cytokinin/auxin ratio is a crucial factor under optimal concentrations of plant growth regulators. Conclusively, the ideal formulation of plant growth regulators for callus induction is 1.1 mg/L of ZT and 0.2 mg/L of NAA.

### 2.3. Effect of Different BA Concentrations on Adventitious Bud Induction

According to the results in Table 3, different BA concentrations significantly influenced both the adventitious bud induction rate and the mean number of buds, with both parameters showing an initial increase followed by a decrease as BA concentrations varied. When the BA concentration was 2 mg/L, the adventitious bud induction rate peaked at 83.33%, accompanied by a mean of 15.17 buds per callus, which were significantly higher than those observed in other treatments, displaying fully expanded leaves and vigorous growth (Figure 2c). Therefore, the optimal BA concentration for adventitious bud induction was determined to be 2 mg/L.

### 2.4. Effects of Different Combinations of BA and NAA Concentrations on Adventitious Shoot Elongation

Table 4 shows that the mean shoot length was significantly affected by different BA and NAA combinations. Notably, the combination of 0.2 mg/L of BA and 0.04 mg/L of NAA demonstrated the optimal effect, yielding a mean shoot length of 1.01 cm. The elongated shoots exhibited robust growth (Figure 2d). In contrast, the 1.0 mg/L of BA and 0.2 mg/L of NAA combination resulted in the shortest mean shoot length of 0.35 cm, with slight leaf curling observed in elongated shoots, indicating the poorest performance. Therefore, the optimal combination for adventitious shoot elongation was determined as 0.2 mg/L of BA and 0.04 mg/L of NAA.

### 2.5. Effect of Different NAA Concentrations on Rooting of Adventitious Shoots

*T. granulosa* exhibited high rooting competence, achieving 100% rooting efficiency across all NAA concentration treatments. However, significant variations in root system architecture were observed between treatments (Table 5). Plantlets treated with the optimal NAA concentration (0.5 mg/L) exhibited significantly enhanced root development, with a mean number of 8.15 roots per plantlet, a root length of 5.03 cm, and a diameter of 0.34 mm. These plantlets developed into robust specimens characterized by fully expanded leaves and sturdy stems (Figure 2e,f). In contrast, the auxin-free control group showed markedly reduced root initiation, averaging only 1.75 roots per plantlet, with a mean root length of 2.63 cm and a mean diameter of 0.27 mm.

### 2.6. Effectiveness of Transplantation

The experimental results confirmed 91.00% of regenerated plantlets survived 30 d post-transplantation. After transplantation, the plantlets exhibited robust growth. (Figure 2g–i).

## 3. Discussion

Indirect organogenesis through callus-mediated regeneration, achieved by culturing various explant organs to induce callus formation followed by adventitious shoot differentiation, enables efficient plantlet regeneration [11]. In Melastomataceae species, such as *Melastoma dodecandrum* [12], *Melastoma affine*, and *Melastoma candidum* [13], MS basal medium has been established as optimal for callus induction. This study corroborates these findings through systematic media screening, demonstrating that the balanced inorganic salt composition in MS medium promotes callus induction. In contrast, elevated salt concentrations, such as those in a 3/2 × MS medium, inhibit callus growth via osmotic stress and ion toxicity [14].

Significant variation in callus induction and differentiation rates existed across explant types, necessitating strategic explant selection to establish efficient regeneration systems [13,15]. This study identifies that the callus induction rate of petiolated leaves was the highest, likely due to the petiole’s lower cellular differentiation state, higher metabolic activity, and enhanced regenerative competence, facilitating rapid dedifferentiation in response to auxin/cytokinin signals [16]. These findings align with observations in *Momordica charantia* [17] and *Pinellia ternate* [18], where petioles consistently outperform other explants in callus formation efficiency. Consequently, petiolated leaf explants represent the optimal choice for de novo organogenesis in *T. granulosa*.

Plant growth regulators play pivotal roles in modulating cellular dedifferentiation and redifferentiation by synergizing with endogenous hormonal pathways to drive callus proliferation and adventitious shoot organogenesis [19]. ZT, a naturally occurring cytokinin, exhibits superior bioactivity compared to kinetin (KT) and BA [20]. Early mechanistic studies demonstrated ZT’s critical involvement in RNA and protein synthesis during organogenesis [21], with subsequent research confirming its capacity to stimulate cell division and differentiation across diverse species [22,23,24]. This study similarly underscores the essential function of ZT in callus induction for *T. granulosato*, with both supraoptimal and suboptimal concentrations adversely affecting callus growth, consistent with findings reported by Meng H.R. [25]. Furthermore, combining ZT with complementary phytohormones enabled direct shoot differentiation from explants [26]. In contrast, Zhang X.H. et al. achieved superior regeneration outcomes in *Tibouchina aspera* leaf cultures using thidiazuron (TDZ), reporting a mean of 11.3 shoots per explant alongside enhanced differentiation rates in subsequent callus induction [27]. Comparable regenerative efficacy of TDZ has also been documented in *T. magnifica* [28] and *Tibouchina urvilleana* [29]. While this study did not investigate TDZ’s effects on *T. granulosato* callus induction or differentiation, future experiments should explore TDZ applications to potentially optimize regeneration efficiency in this species.

In Melastomataceae micropropagation systems, BA is the predominant cytokinin, typically combined with NAA to regulate organogenesis [12,13,15,30,31]. This study demonstrated that all five tested BA concentrations effectively induced adventitious bud formation, contingent upon prior ZT-mediated induction of callus with enhanced differentiation competence. Following shoot induction from callus, shoot elongation can be promoted by reducing phytohormone concentrations. Once transferred to a rooting medium, these elongated shoots develop into vigorous regenerated plantlets [32,33].

## 4. Materials and Methods

### 4.1. Acquisition of Sterile Seedlings

The seeds of *T. granulosa* used in the experiment were collected from the pink-flowered individual plants in the nursery of the Guangzhou Forestry and Landscape Science Research Institute (23.15° N, 113.28° E). Fruits at full maturity (free of pests/disease) were first rinsed with tap water for 15 min to remove surface debris, then sterilized by soaking in 0.1% carbendazim solution for 25 min with constant stirring, followed by rinsing with deionized water 5~6 times to remove residual fungicide. The exocarp was peeled off on an ultra-clean workbench and placed into a sterilized bottle, rinsed once with sterile water, soaked in 75% alcohol for 1 min and 0.1% mercury chloride solution (with 2~3 drops of Tween 20) for 20 min in sequence, with shaking and oscillation taking place at each step, and rinsed 3~5 times with sterile water after each disinfection. Finally, seeds were aseptically excised from the fruits using a sterile scalpel and transferred onto an MS_0_ medium (MS basal medium without plant growth regulators). After 25 d, the aseptic seeds had successfully germinated when the first pair of leaves expanded.

### 4.2. Callus Induction

#### 4.2.1. Explant Selection for Callus Induction

To enhance induction efficiency and obtain structurally well-developed callus, three explants were used: epicotyls, leaves with petioles, and leaves without petioles from 25-d-old aseptic seedlings. Under sterile conditions, the epicotyls were cut into segments of approximately 1 cm; leaves with petioles retained the entire petiole, while leaves without petioles had only the petiole removed. The selected leaf explants were the first pair of fully expanded leaves.

To promote callus formation, explants were subjected to wounding treatments: leaf wounding was performed by orienting perpendicularly to the midrib, excising the leaf tip area, and generating 3–4 epidermal incisions with sterile blades. Epicotyl wounding was achieved through a controlled 2–3 superficial incisions. Explants were cultured adaxial surface downward (leaves) or horizontally (epicotyls) on a callus induction medium.

The medium consisted of MS supplemented with 1.0 mg/L of ZT and 0.3 mg/L of NAA. Each treatment contained 90 explants equally divided into 3 replicates. The callus induction rate and morphological changes in each type of explant were compared after 60 d of culture.

#### 4.2.2. Optimization of PGRs for Callus Induction

A systematic screening of cytokinin–auxin combinations was conducted using petiolated leaf explants (selected as the optimal explant from Section 4.2.1) cultured on MS basal medium. Employing a full factorial design (Table 6), ZT (0.8, 1.0, 1.1, and 1.3 mg/L) and NAA (0.1, 0.2, 0.3, and 0.4 mg/L) were evaluated for synergistic effects on callus induction. Each treatment contained 90 explants equally divided into 3 replicates. Explants were prepared and cultured as described in Section 4.2.1. Callus morphology and induction efficiency were systematically recorded after 60 d.

### 4.3. Adventitious Bud Induction

Preliminary trials utilizing ZT demonstrated effective callus induction; however, its potent cytokinin activity resulted in suboptimal regeneration outcomes characterized by sparse and stunted adventitious buds. To enhance regeneration efficiency, this experiment employed BA in a single-factor design. Green and compact nodular calluses (1.0 cm^3^ cubic blocks) derived from petiolated leaf cultures were aseptically transferred to an MS basal medium supplemented with 0.4 mg/L of NAA and BA gradients (1.3, 1.5, 1.7, 2.0, and 2.3 mg/L). Each treatment contained 90 calluses equally divided into 3 replicates. Calluses were placed horizontally on the medium surface. Adventitious bud induction and the mean number of buds were counted after 25 d.

### 4.4. Elongation of Adventitious Shoots

Calluses with buds were carefully selected for shoot elongation, ensuring a uniform developmental stage (with visible buds and no signs of browning). These calluses were aseptically harvested and transferred to an MS-based elongation medium containing BA and NAA at the concentrations detailed in Table 7. Each treatment contained 90 bud-bearing callus clusters equally divided into 3 replicates. The mean shoot length was measured after 15 d.

### 4.5. Rooting

Elongated shoots with robust stems and fully expanded leaves (≥1 cm in height) were excised and transferred to a 1/2 × MS (half-strength MS macronutrients) basal medium supplemented with gradient concentrations of NAA (0, 0.1, 0.2, 0.3, 0.5, 0.7, and 1.0 mg/L) in a single-factor experimental design. Each treatment contained 90 adventitious shoots equally divided into 3 replicates. The root rate, mean number of roots per plantlet, root length, and root diameter were recorded after 25 d.

### 4.6. Acclimatization and Transplantation

For ex vitro adaptation, strong-rooted plantlets were hardened in a shaded greenhouse (50–80% shade) for 5–7 days, allowing them to adapt to natural light and humidity conditions. They were transplanted into a mix of peat, perlite, and vermiculite (3:1:1 *v*/*v*/*v*), which was pre-sterilized to eliminate pathogenic microorganisms. Sterilization was performed using 0.1–0.2% potassium permanganate solution, left for 24–48 h, rinsed to remove residual disinfectant, and then aired for 1–3 days before use.

During the transplant, plantlets were taken from vessels, and the roots were cleaned of any remaining medium and soaked in 600× carbendazim for 10 min to prevent fungal infections. The soil was gently pressed to conceal the roots. Post-transplant: (1) the area was irrigated well; (2) 600× carbendazim spray was applied for disease prevention; and (3) humidity was maintained with a film for 10 days. After removing the film, standard fertilizing began. A total of 100 vigorous plantlets were selected based on the rooting for transplantation, and their survival was assessed 30 days later.

### 4.7. Culture Condition

The culture medium was supplemented with 8 g/L of carrageenan (Zhao Qing Hai Xing Food Industry Co., Ltd., Guangzhou, China) and 30 g/L of sugar, and the pH value was adjusted to 5.8–6.0 before autoclaving (121 °C, 103 kPa, 20 min). All the above tissue cultures were placed in a constant-temperature, sterile room at 25 ± 2 °C for light culture, with a light intensity of 65 μmol·m^−2^·s^−1^ and a light duration of 12 h/d.

### 4.8. Data Processing

Callus induction rate (%) = number of callus explants induced/number of initial explants × 100. Adventitious bud induction rate = number of calluses with adventitious buds/total number of initial calluses. Mean number of adventitious buds = total number of adventitious buds/total number of calluses with adventitious buds. Mean shoot length = total shoot length/number of initial shoots (with shoot length ≥0.5 cm). Rooting rate (%) = number of rooting plants/number of initial shoots × 100. The mean number of roots per plantlet = total number of roots/number of initial rooted shoots. Mean length of roots per plantlet = total length of roots/total number of roots (from the base of the stem to the root tip). Mean diameter of roots per plantlet = total diameter of roots/total number of roots (5 mm away from the stem). Data were collated and statistically analyzed using IBM SPSS 23.00. The significance of differences among means was determined using Duncan’s Multiple Range Test with a significance level of *p* < 0.05. The results were represented as means ± standard error of three replicates.

## 5. Conclusions

This study established a regeneration system for *T. granulosa* for the first time. The optimized protocols demonstrate that petiolated leaves achieve optimal callus induction on MS medium supplemented with 1.1 mg/L of ZT and 0.2 mg/L of NAA, while adventitious bud induction is most effective when containing 2.0 mg/L of BA and 0.4 mg/L of NAA. Rooting is successfully promoted with the addition of 0.5 mg/L NAA. The establishment of this robust regeneration system will significantly accelerate the adoption of *T. granulosa* cultivation in China and serve as a vital foundation for future development of novel cultivars.

## Figures and Tables

**Table 1:** Effect of explant types on callus induction.

Explant Type	Callus Induction Rate (%)	Callus Morphology
Epicotyl	70.83 ± 2.41 A	Yellow–green
Petiolated leaf	75.00 ± 2.41 A	Green, compact
Petiole-free leaf	56.94 ± 3.67 B	Green

Notes: Values represent mean ± SE. Capital letters indicate significant differences (*p* < 0.05) by Duncan’s multiple range test.

**Table 2:** Effect of ZT and NAA concentration combinations on callus induction.

NO.	ZT (mg/L)	NAA (mg/L)	Callus Induction Rate (%)	Callus Morphology
1	0.8	0.1	39.58 ± 2.08 HI	Yellow–green, severe browning, and partial whitening
2	0.8	0.2	45.84 ± 1.04 GH	Yellow–green, moderate browning, and partial whitening
3	0.8	0.3	44.79 ± 2.75 GH	Sporadic adventitious roots, moderate browning, and partial whitening
4	0.8	0.4	31.25 ± 0.00 I	Sporadic adventitious roots, moderate browning, and partial whitening
5	1.0	0.1	53.13 ± 4.77 FG	Green and compact nodular
6	1.0	0.2	77.09 ± 3.76 BC	Green and compact nodular
7	1.0	0.3	69.79 ± 1.04 CD	Green and compact nodular
8	1.0	0.4	45.84 ± 2.75 GH	Green and nodular
9	1.1	0.1	65.63 ± 5.41 DE	Green and compact nodular
10	1.1	0.2	89.59 ± 3.76 A	Green and compact nodular
11	1.1	0.3	81.25 ± 1.80 AB	Green and compact nodular
12	1.1	0.4	77.08 ± 4.17 BC	Green and nodular
13	1.3	0.1	56.25 ± 3.61 EF	Yellow–green and partly browned
14	1.3	0.2	71.88 ± 1.80 BCD	Yellow–green and partly browned
15	1.3	0.3	57.29 ± 4.54 EF	Severe browning and partial whitening
16	1.3	0.4	38.54 ± 2.75 HI	Severe browning and partial whitening

Notes: Values represent mean ± SE. Capital letters indicate significant differences (*p* < 0.05) by Duncan’s multiple range test.

**Table 3:** Effect of different BA concentrations on adventitious bud induction.

BA (mg/L)	Adventitious Bud Induction Rate (%)	Mean Number of Buds
1.3	51.11 ± 2.94 C	2.82 ± 0.17 D
1.5	67.78 ± 4.84 B	5.43 ± 0.11 C
1.7	72.22 ± 2.94 AB	7.96 ± 0.15 B
2.0	83.33 ± 3.85 A	15.17 ± 0.42 A
2.3	70.00 ± 3.33 B	5.96 ± 0.25 C

Notes: Values represent mean ± SE. Capital letters indicate significant differences (*p* < 0.05) by Duncan’s multiple range test.

**Table 4:** Effects of different combinations of BA and NAA concentrations on adventitious buds’ elongation.

6-BA (mg/L)	NAA (mg/L)	Mean Shoot Length (cm)
1.0	0.2	0.35 ± 0.013 B
0.2	0.04	1.01 ± 0.041 A
0.1	0.01	0.42 ± 0.021 B

Notes: Values represent means ± SE. Capital letters indicate significant differences (*p* < 0.05) in Duncan’s multiple range test.

**Table 5:** Effect of different NAA concentrations on rooting of adventitious shoots.

NAA (mg/L)	Mean Number of Roots per Plantlet	Mean Length of Roots per Plantlet	Mean Diameter of Roots per Plantlet
0	1.75 ± 0.20 F	2.63 ± 0.19 D	0.27 ± 0.01 C
0.1	3.82 ± 0.17 E	3.13 ± 0.15 CD	0.31 ± 0.02 B
0.2	4.59 ± 0.14 D	3.53 ± 0.18 BC	0.31 ± 0.01 AB
0.3	6.90 ± 0.26 B	4.67 ± 0.12 A	0.32 ± 0.01 AB
0.5	8.15 ± 0.30 A	5.03 ± 0.33 A	0.34 ± 0.00 A
0.7	6.27 ± 0.19 BC	3.97 ± 0.17 B	0.32 ±0.00 AB
1.0	5.60 ± 0.38 C	3.57 ± 0.18 BC	0.31 ± 0.01 AB

Notes: Values represent mean ± SE. Capital letters indicate significant differences (*p* < 0.05) by Duncan’s multiple range test.

**Table 6:** Full factorial design for callus induction.

ZT (mg/L)	NAA (mg/L)	ZT (mg/L)	NAA (mg/L)
0.8	0.1	1.1	0.1
0.8	0.2	1.1	0.2
0.8	0.3	1.1	0.3
0.8	0.4	1.1	0.4
1.0	0.1	1.3	0.1
1.0	0.2	1.3	0.2
1.0	0.3	1.3	0.3
1.0	0.4	1.3	0.4

**Table 7:** Different combinations of BA and NAA concentrations.

BA (mg/L)	NAA (mg/L)
1.0	0.2
0.2	0.04
0.1	0.01

## Data Availability

The data supporting the results of this report are available in the attachment.
